# Reaching the Monophyly: Re-Evaluation of the Enigmatic Species *Tenuibiotus hyperonyx* (Maucci, 1983) and the Genus *Tenuibiotus* (Eutardigrada)

**DOI:** 10.3390/ani12030404

**Published:** 2022-02-08

**Authors:** Daniel Stec, Witold Morek

**Affiliations:** 1 Institute of Systematics and Evolution of Animals, Polish Academy of Sciences, Sławkowska 17, 31-016 Kraków, Poland; 2 Department of Invertebrate Evolution, Institute of Zoology and Biomedical Research, Faculty of Biology, Jagiellonian University, Gronostajowa 9, 30-387 Kraków, Poland; wmorek@op.pl

**Keywords:** *Diaforbiotus*, Dolomite Alps, integrative taxonomy, Macrobiotoidea, Tardigrada, taxonomic revision

## Abstract

**Simple Summary:**

The tardigrade species *Tenuibiotus hyperonyx* (Maucci, 1982) was described forty years ago from the highest mountain of the Sella Group within the Dolomites (Italy), and so far, it is known only from its type locality. Due to the morphological characters of the claws, it has been included in the genus *Tenuibiotus* (Pilato & Lisi, 2011). By conducting the integrative and phylogenetic analyses, we pinpointed the phylogenetic position of the species, which turned out to be positioned within the genus *Diaforobiotus* Guidetti et al., 2016. As the phenotypic characters of the examined species fit the diagnosis of this genus, we proposed a new combination, *Diaforobiotus hyperonyx* (Maucci, 1982) comb. nov. Furthermore, we looked more closely for the morphological diversity noted within the genus *Tenuibiotus*, clarifying phenotypic uncertainties regarding *Tenuibiotus willardi* (Pilato, 1977) and *Tenuibiotus bozhkae* Pilato, Kiosya, Lisi, Inshina & Biserov, 2011. This action leads to uniformization of the genus diagnosis from the morphological point of view, which brings it closer to being considered as monophyletic.

**Abstract:**

Revisions and redescriptions of taxa described in the past and that are now categorized as insufficiently diagnosed often play a crucial role in making further progress in modern taxonomy in many groups of organisms. Here we revised an enigmatic tardigrade species *Tenuibiotus hyperonyx* (Maucci, 1983) based on the newly discovered topotypic population from the Italian Alps. We performed an integrative analysis of morphological and genetic data in order to present an upgraded species description and elucidate its phylogenetic position. Our results enabled us to confidently place *T.* *hyperonyx* within the family Richtersiusidae, as a member of the genus *Diaforobiotus*. This change, together with a re-assessment of microphotographs of the *Tenuibiotus willardi* (Pilato, 1977) and *Tenuibiotus bozhkae* Pilato, Kiosya, Lisi, Inshina & Biserov, 2011 types, led to the discussion on species composition with narrative taxa amendments for the taxonomic parties involved in the proposed alteration.

## 1. Introduction

Tardigrades are microinvertebrates (body size rarely exceeding 1 mm) found mostly in mosses and lichens [1]. The phylum comprises almost 1400 species; however, the real number of taxa that can be recognized in this group seems much higher, as each year brings dozens of new species that are being described and formally named [2,3,4]. Over the last ten years, the implementation of molecular techniques and taxonomic analyses by means of an integrative approach have accelerated the emergence of new species being characterized morphologically and genetically in detail, e.g., [5,6,7,8,9,10,11,12]. The acquisition and accumulation of genetic data tightly associated with phenotypic information enhanced studies on two major subjects in tardigrade taxonomy: (i) The recognition and disentanglement of cryptic/pseudocryptic diversity, e.g., [13,14,15,16,17,18,19] and (ii) the construction of comprehensive molecular phylogenies at the family level or higher that considerably impacted tardigrade systematics, e.g., [20,21,22,23,24,25,26,27,28,29,30]. Notably, there were not only integrative descriptions of new species for science that have contributed greatly to these subjects’ investigation, but also revisional notes with updated diagnoses and new information on already-known taxa, e.g., [31,32,33,34,35,36,37]. All these contributions explicitly underline the importance of being integrative when studying tardigrade systematics [38,39].

*Macrobiotus hyperonyx* Maucci, 1982 [40] was described from the highest mountain of the Sella Group (Piz Boè, 3152 m a.s.l) within the Dolomites (Italy), and so far, it is known only from its type locality. Due to the animal’s morphology, it has been included within the informal *tenuis*-group by Maucci [41,42], which was later revised by Tumanov [43,44]. The morphological premises of the *tenuis*-type claw (a common tract distinctly longer compared to other macrobiotids and a short secondary branch forming an almost right angle with the primary branch) have been considered as a main diagnostic characteristic for the genus *Tenuibiotus* erected by Pilato and Lisi [45]. The genetic data for the genus *Tenuibiotus* are limited to the four taxa that were published in three scientific papers [29,46,47]. Only in the first work did the authors demonstrate the monophyly of these taxa in their extensive phylogenetic study on the family Macrobiotidae. However, it was noted that all these *Tenuibiotus* populations were morphologically uniform with a non-porous cuticle and two macroplacoids in the pharynx [29]. Therefore, as some of the taxa originally attributed to the genus exhibit pores in the cuticle or three macroplacoids in the pharynx, and these traits are known to have great importance for eutardigrade classification, Stec et al. [29] suggested *Tenuibiotus* with such taxa composition to be polyphyletic.

In this work, we rediscovered a topotypic population of *Tenuibiotus hyperonyx* (Maucci, 1982) [40] in the Dolomite Alps on which the integrative taxonomic analysis was performed. This included detailed morphological and morphometric examination under phase contrast and scanning electron microscopy (PCM and SEM, respectively) and sequencing of four molecular markers (18S rRNA, 28S rRNA, ITS-2 and COI). Given that *T. hyperonyx* exhibits pores in the body cuticle, we were able to test the hypothesis on *Tenuibiotus* polyphyly by elucidating its phylogenetic position. Additionally, the examination of microphotographs of the *Tenuibiotus willardi* (Pilato, 1977) [48] types enabled us to verify the presence of taxa possessing three macroplacoids in the pharynx within the genus.

## 2. Material and Methods

### 2.1. Samples and Specimens

Four moss or moss + lichen samples containing *T. hyperonyx* were collected from rocks at the nival zone in Trento (the Dolomites, Piz Boè; all at ca. 3000 m a.s.l.) by Witold Morek and Katarzyna Vončina on 18 July 2020 (Table 1). The samples were examined for tardigrades according to Stec et al. [49]. Other tardigrades present in the samples included *Cornechiniscus holmeni* (Petersen, 1951) [50], *Echiniscus granulatus* (Doyère, 1840) [51], and representatives of the genera *Milnesium*, *Pseudechiniscus*, and *Richtersius*. Extracted animals and eggs were divided into several groups used for different analyses, i.e., morphological analysis in PCM and SEM, as well as DNA sequencing (Table 1). Two additional specimens of *Crenubiotus* sp. from a Greenlandic moss sample (Sermersooq; 74°29′0.766″ N, 20°32′18.308″ W; 77 m a.s.l.; moss from soil; tundra; 07.2021; coll. Michał Kolasa) were sequenced in order to increase our phylogenetic dataset.

### 2.2. DNA Sequencing

DNA was extracted from individual animals following a Chelex^®^ 100 resin (BioRad, Warsaw, Poland) extraction method by Casquet et al. [52] with modifications described in detail in Stec et al. [35]. Each specimen was mounted in water and examined under light microscopy prior to DNA extraction. We sequenced four DNA fragments, three nuclear (18S rRNA, 28S rRNA, ITS2) and one mitochondrial (COI). All fragments were amplified and sequenced according to the protocols described in Stec et al. [35]; primers with original references are listed in Table 2. Sequencing products were read with the ABI 3130xl sequencer at the Molecular Ecology Lab, Institute of Environmental Sciences of the Jagiellonian University, Kraków, Poland. Newly obtained sequences were submitted to NCBI GenBank (see “Results” section and Table 3). Sequences in this study were processed and handle in BioEdit ver. 7.2.5 [53].

### 2.3. Phylogenetic Analyses

For phylogenetic analyses, we used a dataset that comprises concatenated DNA sequences of 18S rRNA + 28S rRNA + ITS-2 + COI markers. The BLAST search [67] with newly generated sequences as a query recovered their highest similarity with the Richtersiusidae Guidetti, Schill, Giovannini, Massa, Goldoni, Ebel, Förschler, Rebecchi & Cesari, 2021 [11] taxa. Therefore, the phylogenetic dataset comprised taxa analyzed in Stec et al. [27] when erecting the family Adorybiotidae Stec, Vecchi & Michalczyk, 2020 [27]. The dataset was supplemented with additional sequences of (i) Adorybiotidae and Richtersiusidae published after 2020, (ii) additional sequences of Murrayidae Guidetti, Rebecchi & Bertolani, 2000 [68] and Eohypsibidae Bertolani & Kristensen, 1987 [69] available in GenBank but unintentionally omitted in Stec et al. [27], as well as (iii) DNA sequences newly obtained in this study. Sequences were downloaded from GenBank, and the full list of accession numbers is given within Table 3. 

The sequences were aligned using the AUTO method (in the case of COI and ITS-2) and the Q-INS-I method (18S rRNA and 28S rRNA) in MAFFT version 7 [70,71] and manually checked against non-conservative alignments in BioEdit. Then, the aligned sequences were trimmed to 1009 (18S rRNA), 832 (28S rRNA), 543 (ITS-2) and 658 (COI) bp. All COI sequences were translated into protein sequences in MEGA7 version 7.0 [72] to check against pseudogenes. The sequences were then concatenated in SequenceMatrix [73]. Using PartitionFinder version 2.1.1 [74] under the Akaike Information Criterion (AIC), and with a greedy algorithm [75] implemented within the software, we chose the best scheme of partitioning and substitution models for posterior phylogenetic analysis. We ran the analysis to test all possible models implemented in MrBayes and RAxML software. As the COI is a protein-coding gene, before partitioning, we divided our alignment of this marker into three data blocks constituting three separated codon positions.

Bayesian inference (BI) marginal posterior probabilities were calculated using MrBayes v3.2 [76]. Random starting trees were used, and the analysis was run for 10 million generations, sampling the Markov chain every thousand generations. An average standard deviation of split frequencies of <0.01 was used as a guide to ensure the two independent analyses had converged. The program Tracer v1.6 [77] was then used to ensure Markov chains had reached stationarity and to determine the correct ‘burn-in’ for the analysis, which was the first 10% of generations. The ESS values were greater than 200 and a consensus tree was obtained after summarizing the resulting topologies and discarding the ‘burn-in’. The maximum-likelihood (ML) tree was computed using RAxML v8.0.19 [78]. The strength of support for internal nodes of the ML construction was measured using 1000 rapid bootstrap replicates. All final consensus trees were visualized with FigTree v.1.4.3 available from (http://tree.bio.ed.ac.uk/software/figtree, accessed on 10 August 2018).

### 2.4. Microscopy and Imaging

Specimens for light microscopy were mounted on microscope slides following the protocol by Morek et al. [79]. Slides were examined under an Olympus BX53 light microscope with phase contrast (PCM), associated with an Olympus DP74 digital camera. Immediately after mounting the specimens in the medium, slides were checked under PCM for the presence of males and females in the studied population [61,80]. Specimens for the SEM analysis were processed according to the protocol by Stec et al. [49]. Bucco-pharyngeal apparatuses were extracted following the protocol of Eibye-Jacobsen [81] as modified by Gąsiorek et al. [82]. Specimens were examined under high vacuum in a Versa 3D DualBeam Scanning Electron Microscope (SEM) at the ATOMIN facility of the Jagiellonian University, Kraków, Poland. All figures were assembled in Corel Photo-Paint X6.

### 2.5. Morphometry and Morphological Nomenclature

All measurements are given in micrometers (μm). The sample size was adjusted following recommendations in Stec et al. [83]. Structures were measured only if their orientation was suitable. Body length was measured from the anterior extremity to the end of the body, excluding the hind legs. The terminology used to describe the oral cavity armature and egg-shell morphology follows Michalczyk and Kaczmarek [84] and Kaczmarek and Michalczyk [85]. The macroplacoid length sequence is given according to Kaczmarek et al. [86] whereas morphological states of the cuticular bars on legs follow Kiosya et al. [36]. The buccal tube length and the level of the stylet support insertion point were measured according to Pilato [87]. The *pt* index was calculated as the ratio of the length of a given structure to the length of the buccal tube expressed as a ratio [87]. Measurements of buccal tube widths, heights of claws heights and eggs follow Kaczmarek and Michalczyk [85]. The claw common tract index (cct) is the proportion of the height of the common tract of the claw (measured from the claw base to the separation point between the first and the second branch) to the total claw height expressed as a percentage [22]. Morphometric data were handled using the “Parachela” ver. 1.8 template available from the Tardigrada Register [88]. Eutardigrade taxonomy follows [11,20,27,29].

### 2.6. Comparative Material

Microphotographs of animals and eggs from the type series of *T. hyperonyx* from the Maucci collection (Civic Museum of Natural History of Verona, Verona, Italy) were kindly provided by Denis Tumanov. Additional microphotographs of the *T. hyperonyx* types were taken by Witold Morek and Piotr Gąsiorek during their visit in 2017 to the Evolutionary Zoology Lab (Department of Life Sciences, University of Modena and Reggio Emilia). Microphotographs of animals and eggs from the type series of *T. willardi* from the Pilato and Binda collection as well as the Bertolani collection were kindly provided by Oscar Lisi and Matteo Vecchi, respectively. Microphotographs of the holotype and the paratype of *Tenuibiotus bozhkae* Pilato, Kiosya, Lisi, Inshina & Biserov, 2011 [89] from the Pilato and Binda collection were kindly provided by Oscar Lisi.

### 2.7. Availability of Data and Materials

DNA sequences for the examined populations are deposited in GenBank (https://www.ncbi.nlm.nih.gov/genbank, accessed on 10 August 2018). Best-fit partitioning schemes and models suggested by PartitionFinder are given within Supplementary Materials SM.01. Raw Bayesian and Maximum Likelihood trees are given in the Newick format within Supplementary Materials SM.02. Raw morphometric measurements of the newly discovered topotypic population of *T. hyperonyx* are given in Supplementary Materials SM.03. A movie recording of an alive, gravid female of *T. hyperonyx* is given in Supplementary Materials SM.04.

## 3. Results

### 3.1. Phylogenetic Position of T. hyperonyx

**Figure 1:**
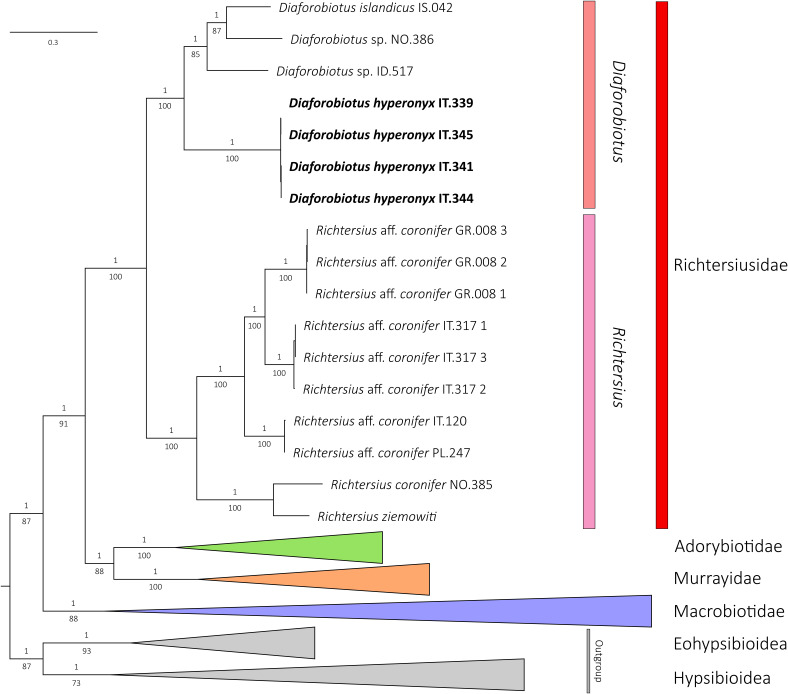
Phylogenetic reconstruction of the superfamily Macrobiotoidea based on concatenated 18S rRNA + 28S rRNA + ITS-2 + COI nucleotide sequences. Topology and branch length of BI reconstruction. Values above branches indicate BI posterior probabilities, values below branches indicate ML bootstrap support. Supports for intraspecific nodes are not shown. Newly sequenced specimens of *D. hyperonyx* **comb. nov.** are indicated by a bolded font.

The phylogenetic reconstructions performed with BI and ML methods showed identical topologies, with well-supported nodes in each final tree (Figure 1). The monophyletic superfamily Macrobiotoidea was represented by four well-supported clades representing four valid and monophyletic families: Macrobiotidae, Murrayidae, Adorybiotidae and Richtersiusidae. Specimens of *Tenuibiotus hyperonyx* analyzed in this study have been recovered as a member of the genus *Diaforobiotus* Guidetti et al., 2016 [22] staying in a sister relationship with all other *Diaforobiotus* species (Figure 1). Other *Tenuibiotus* taxa analyzed in this study have been recovered as valid members of the family Macrobiotidae (Supplementary Materials SM.02). Thus, by the placement of *T. hyperonyx* within the family Richtersiusidae and its morphological similarity to the genus *Diaforobiotus,* the species is further transferred and proposed with a new nomenclatural combination as follows: *Diaforobiotus hyperonyx* **comb. nov.** (Figure 1; see the next sections below for details). All *Crenubiotus* Lisi, Londoño & Quiroga, 2020 [90] taxa, including the newly analyzed Greenlandic population, formed a well-supported clade within the family Adorybiotidae in the BI and ML analyses (Supplementary Materials SM.02).

### 3.2. Amended Description of D. hyperonyx **comb. nov.**

#### 3.2.1. Systematic and Taxonomic Account

**Phylum:** Tardigrada Doyère, 1840 [51].

**Class:** Eutardigrada Richters, 1926 [91].

**Order:** Parachela Schuster et al., 1980 [92].

**Superfamily:** Macrobiotoidea Thulin, 1928 [93].

**Family:** Richtersiusidae Guidetti, Schill, Giovannini, Massa, Goldoni, Ebel, Förschler, Rebecchi & Cesari, 2021 [11].

**Genus:** *Diaforobiotus* Guidetti, Rebecchi, Bertolani, Jönsson, Kristensen & Cesari, 2016 [22].

*Diaforobiotus hyperonyx* **comb. nov.** (Maucci, 1982) [40] (Table 4 and Table 5, Figure 2, Figure 3, Figure 4, Figure 5, Figure 6, Figure 7, Figure 8, Figure 9, Figure 10, Figure 11, Figure 12 and Figure 13).

#### 3.2.2. Material Examined 

In total, we examined 64 animals and 4 eggs. Specimens were mounted on microscope slides in Hoyer’s medium (46 animals + 3 eggs), fixed on SEM stubs (14 + 1, including four bucco-pharyngeal apparatuses) and processed for DNA sequencing (4 animals); details on topotypic locality and specific samples are given in Table 1 and the “Material and Methods” section.

#### 3.2.3. Slide and SEM Stubs Depositories

Slides containing 24 animals and 2 eggs (from samples: IT.339 and IT.344) are deposited at the Institute of Systematics and Evolution of Animals (PAS); slides containing 22 animals and 1 egg (from samples: IT.341 and IT.345) and SEM stubs are deposited at the Institute of Zoology and Biomedical Research (JU).

**Figure 2:**
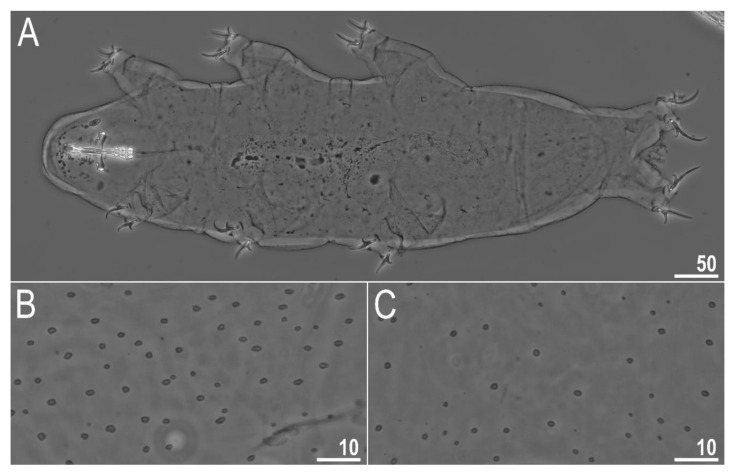
*Diaforobiotus hyperonyx***comb. nov.** (Maucci, 1982): Habitus and cuticular pores seen in PCM: (**A**) Adult habitus, dorso-ventral projection; (**B**,**C**) cuticular pores on dorsal and ventral side of the body, respectively. Scale bars in μm.

**Figure 3:**
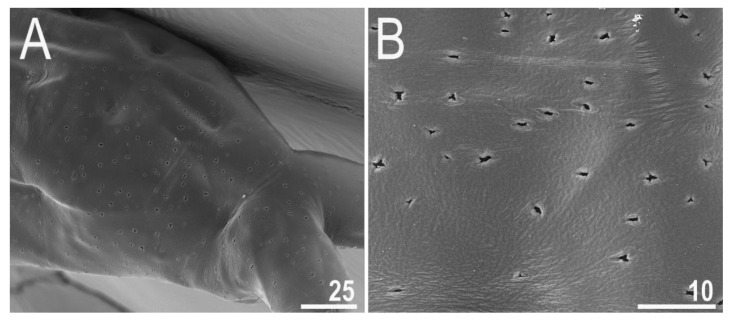
*Diaforobiotus hyperonyx***comb. nov.** (Maucci, 1982): (**A**,**B**) Cuticular pores on dorsal side of the body seen in SEM. Scale bars in μm.

#### 3.2.4. Animals

**Figure 4:**
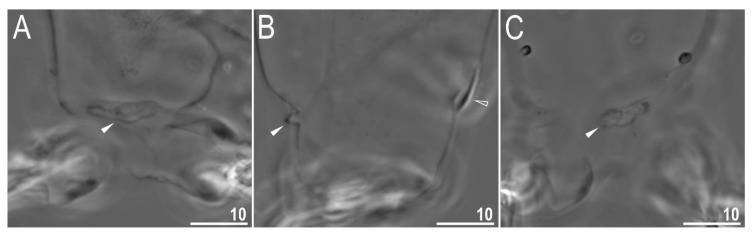
*Diaforobiotus hyperonyx***comb. nov.** (Maucci, 1982): Leg cuticular structures seen in PCM: (**A**) Cuticular fold on the frontal surface of leg III; (**B**) optical midsection of leg II; (**C**) cuticular fold on the frontal surface of leg IV. Filled flat arrowheads indicate cuticular fold whereas empty flat arrowhead indicates cuticular bare above the claws. Scale bars in μm.

**Figure 5:**
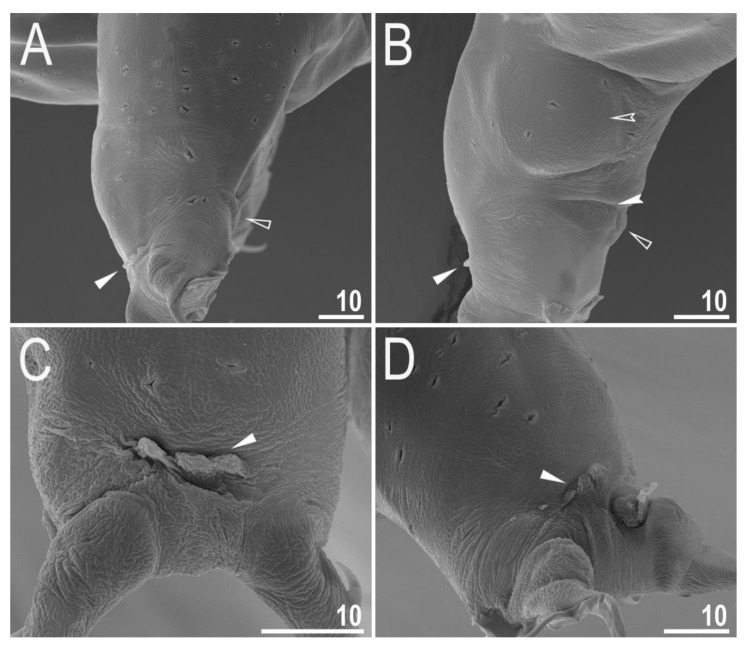
*Diaforobiotus hyperonyx***comb. nov.** (Maucci, 1982): Leg cuticular structures seen in SEM: (**A**,**B**) Lateral view on the external and internal surface of leg II, respectively; (**C**) cuticular fold on the frontal surface of leg II; (**D**) cuticular fold on the frontal surface of leg IV. Filled flat arrowheads indicate cuticular fold, empty flat arrowheads indicate cuticular bare above the claws, filled indented arrowhead indicates muscle attachment above the cuticular bare whereas empty indented arrowhead indicates pulvinus. Scale bars in μm.

When alive, body pale yellow to light orange (Supplementary Materials SM.04); after fixation in Hoyer’s medium, body transparent (Figure 2A). Large, black granular eyes present, also visible in specimens mounted in Hoyer’s medium. Body cuticle smooth, without granulation but with circular or elliptical pores with uneven edges (0.8–2.0 µm in diameter) distributed randomly on the entire body cuticle with the largest pores present in the dorso-caudal cuticle (Figure 2B,C and Figure 3A,B). Pores on the ventral side of the body less frequent than on the dorsal side (Figure 2B,C). Granulation on all legs absent (Figure 4A–C and Figure 5A–D). An obvious cuticular fold is present on the frontal side of each leg I–IV and clearly visible in PCM and SEM (Figure 4A–C and Figure 5A–D). The pulvini are present on each leg I–III on the internal leg surface and are almost indistinct in PCM but clearly visible in SEM (Figure 5B).

**Figure 6:**
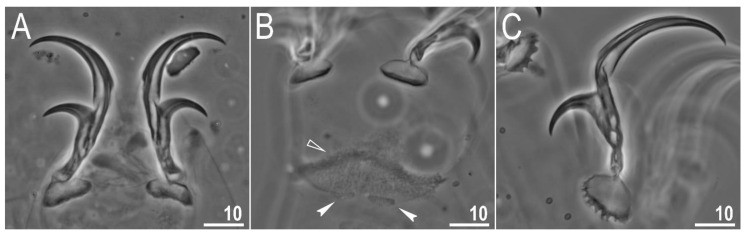
*Diaforobiotus hyperonyx***comb. nov.** (Maucci, 1982): Claws seen in PCM: (**A**) Claws III; (**B**) lunulae of claws III and cuticular bar; (**C**) claws IV. Empty flat arrowhead indicates cuticular bare above the claws whereas filled indented arrowheads indicate double muscle attachments. Scale bars in μm.

**Figure 7:**
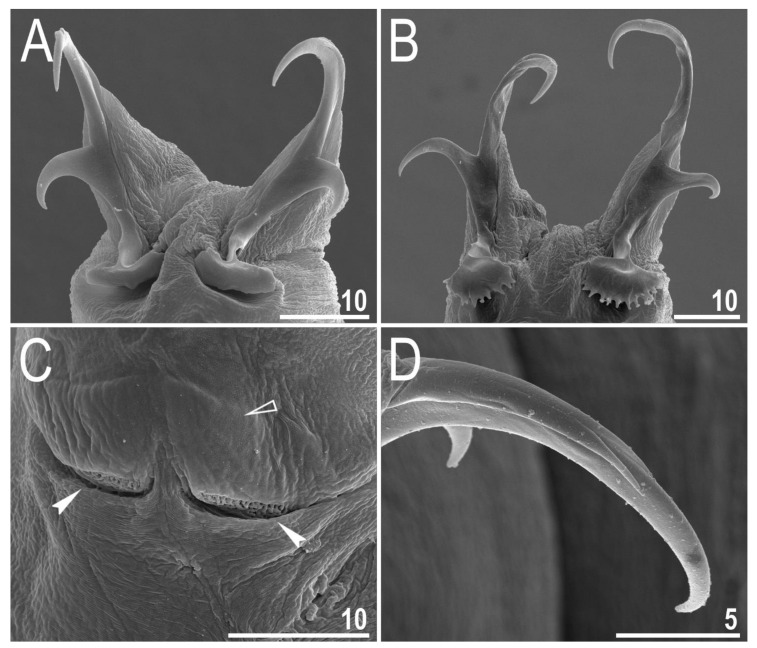
*Diaforobiotus hyperonyx***comb. nov.** (Maucci, 1982): Claws seen in SEM: (**A**) Claws III; (**B**) claws IV; (**C**) cuticular bar and double muscle attachments above the claws; (**D**) details of primary claw branch and accessory points morphology. Empty flat arrowhead indicates cuticular bare above the claws whereas filled indented arrowheads indicate double muscle attachments. Scale bars in μm.

Claws slender, of the richtersiusid type. Common tract with a system of internal septa, and with an evident stalk connecting the claw to the lunula (Figure 6A–C and Figure 7A,B). The common tract nearly as long as the half of the entire claw height (Figure 6A,C and Figure 7A,B). Primary and secondary branches form almost a right angle when bifurcating (Figure 6A,C and Figure 7A,B). Primary branches with accessory points fitted tightly to the branch and thus indistinct in PCM but well visible only in SEM (Figure 6A,C and Figure 7A,B). Large, wide lunulae present on all legs and only in hind legs equipped with clearly visible teeth (Figure 6A–C and Figure 7A,B). A single continuous cuticular bar and paired muscle attachments present just above claws on legs I–III (Figure 4B, Figure 5A,B, Figure 6B and Figure 7C). In PCM, the cuticular bar is indented towards the lunulae, with shaded extensions towards muscle attachments, whereas in SEM, it is visible as a continuous (only sometimes constricted in the middle) thickening (Figure 5A,B, Figure 6B and Figure 7C).

**Figure 8:**
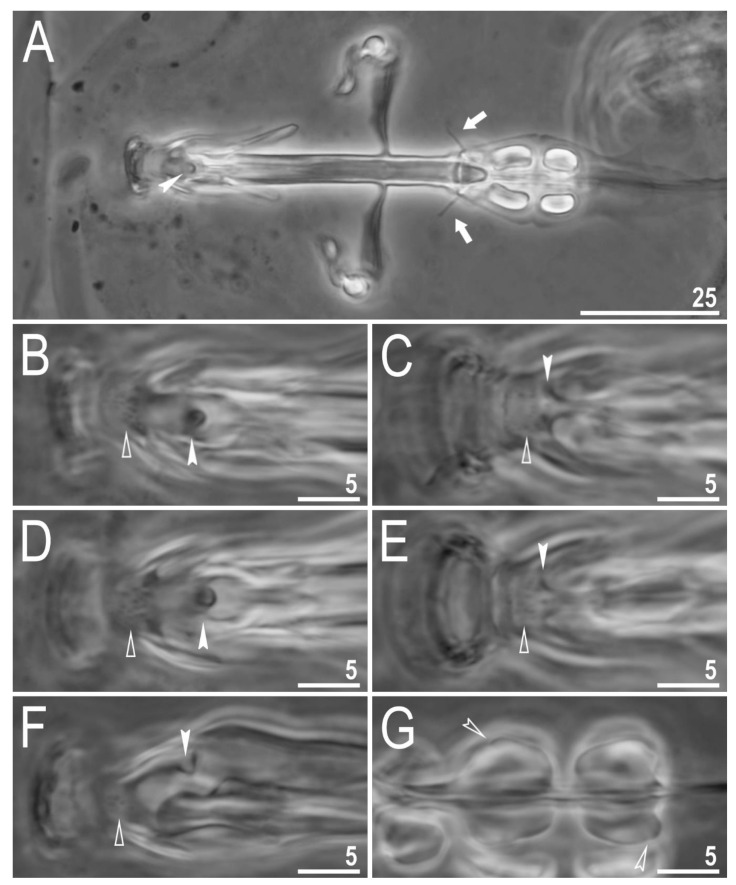
*Diaforobiotus hyperonyx***comb. nov.** (Maucci, 1982): Bucco-pharyngeal apparatus seen in PCM: (**A**) Dorsal projection of the entire buccal apparatus; (**B**–**E**) dorsal (**B**,**D**) and ventral (**C**,**E**) views of the oral cavity armature of two different specimens; (**F**) lateral view of the anterior portion of the bucco-pharyngeal apparatus; (**G**) ventral view of macroplacoids. Arrows indicate dorsal spikes, empty flat arrowheads indicate the second band of teeth, filled indented arrowheads indicate the third band of teeth whereas empty indented arrowheads indicate constrictions in macroplacoids. Scale bars in μm.

**Figure 9:**
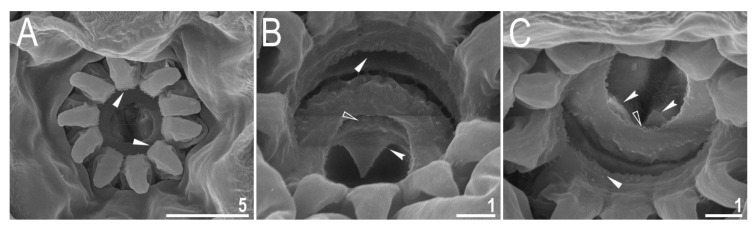
*Diaforobiotus hyperonyx***comb. nov.** (Maucci, 1982): Oral cavity seen in SEM: (**A**) General view of the mouth opening; (**B**,**C**) dorsal and ventral views of the oral cavity armature seen from different angles. Filled flat arrowheads indicate the first band of teeth, empty flat arrowheads indicate the second band of teeth whereas filled indented arrowheads indicate the third band of teeth. Scale bars in μm.

**Figure 10:**
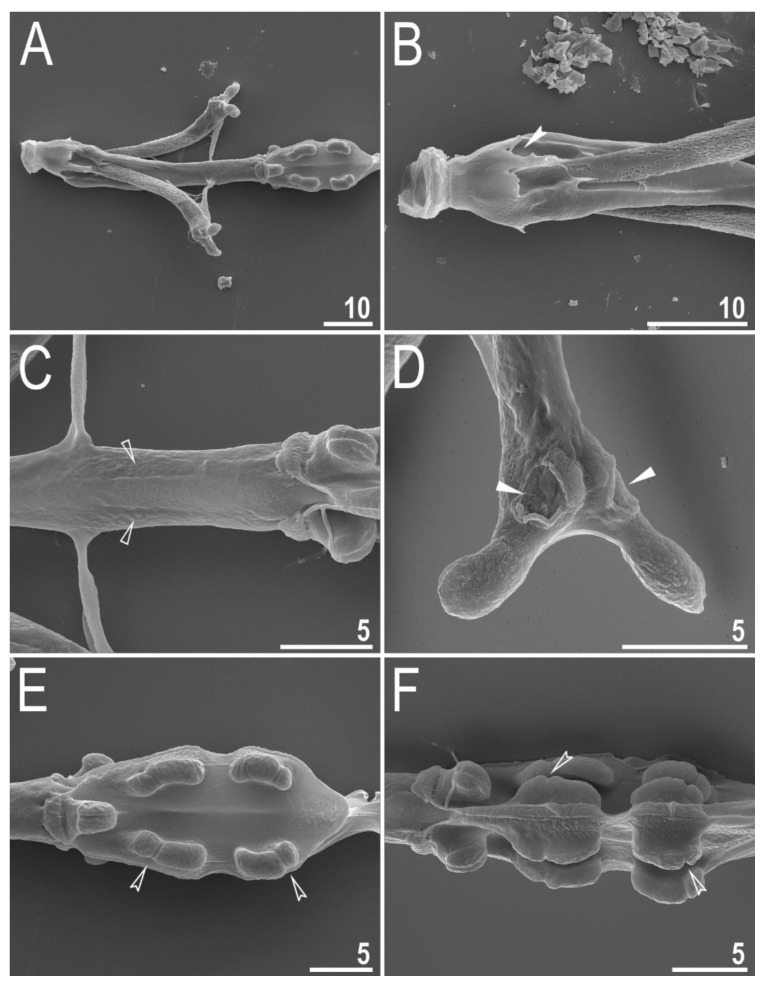
*Diaforobiotus hyperonyx***comb. nov.** (Maucci, 1982): Bucco-pharyngeal apparatus seen in SEM: (**A**) General view of buccal apparatus; (**B**) details of buccal crown; (**C**) details of posterior portion of the buccal tube, ventral view; (**D**) stylet furca; (**E**,**F**) pharynx with macroplacoids. Filled indented arrowhead indicates perforated area in the buccal crown, empty flat arrowheads indicate depressions in the buccal tube below the stylet support insertion points, filled flat arrowheads indicate depressed circular areas in the basal portion of the stylet furca whereas empty indented arrowheads indicate constrictions in macroplacoids. Scale bars in μm.

Mouth antero-ventral. Relatively short bucco-pharyngeal apparatus (Figure 8A and Figure 10A) with ten peribuccal lamellae, a rigid buccal tube, bended anteriorly, with the ventral lamina. Based on PCM observations, the oral cavity armature is poorly developed and composed only of the second and the third band of teeth (Figure 8B–F). However, the first band is present and visible only in SEM and composed of very small granular teeth positioned just below peribuccal lamellae (Figure 9A–C). In PCM, as well as in SEM, the second band of teeth is composed of several rows of granular teeth, of which the most posterior row comprises the larger teeth (Figure 8B–E and Figure 9B,C). The teeth of the third band are located within the posterior portion of the oral cavity, anteriorly to the buccal tube opening (Figure 8B–F and Figure 9A–C). The third band of teeth is divided into the dorsal and the ventral portion (Figure 8B–F and Figure 9A–C). The dorsal portion is composed of only one large tooth positioned in the very posterior portion of the oral cavity and far from the second band of teeth, whereas the ventral portion comprises small lateral ridges, between which a minute medial tooth is present (Figure 8B–E and Figure 9B,C). The ventral portion of the third band of teeth is especially faint in PCM (Figure 8C,E). The porous areas are present in the buccal crown (Figure 10B). In SEM, two depressions are visible on the ventral side of the buccal tube just below the stylet support insertion points (Figure 10C). Typically shaped furcae with enlarged basal portion also exhibiting two depressed circular areas just above the two caudal branches (visible only in SEM; Figure 10D). Pharynx spherical, with triangular apophyses, three anterior cuticular spikes (typically only two are visible in any given plane) and two rod-shaped macroplacoids (2 < 1) (Figure 8G and Figure 10E,F). The first macroplacoid is anteriorly narrowed and constricted in the middle, whereas the second has a sub-terminal constriction (Figure 8G and Figure 10E,F). Microplacoid absent. Measurements of animals and statistics are presented in Table 4.

**Table 4:** Measurements (in µm) of selected morphological structures of animals from the topotypic population of *D. hyperonyx* **comb. nov.** (Maucci, 1982) mounted in Hoyer’s medium; N—number of specimens/structures measured, RANGE refers to the smallest and the largest structure among all measured specimens; SD—standard deviation.

Character	N	Range		Mean		SD	
		µm	*pt*	µm	*pt*	µm	*pt*
Body length	18	449–961	*1095*–*1888*	663	*1394*	137	*186*
Buccal tube							
Buccal tube length	18	34.8–52.6		47.2		5.1	
Stylet support insertion point	18	25.8–39.3	*72.0*–*74.7*	34.6	*73.3*	3.8	*0.9*
Buccal tube external width	18	3.7–6.1	*10.2*–*11.8*	5.2	*10.9*	0.7	*0.5*
Buccal tube internal width	18	2.0–3.3	*4.6*–*6.5*	2.7	*5.7*	0.4	*0.6*
Ventral lamina length	17	19.0–28.7	*49.1*–*56.6*	25.7	*53.6*	2.7	*2.5*
Placoid lengths							
Macroplacoid 1	18	5.4–9.9	*12.7*–*19.4*	7.5	*15.8*	1.1	*1.3*
Macroplacoid 2	18	4.0–8.7	*11.4*–*16.5*	6.1	*12.8*	1.1	*1.1*
Macroplacoid row	18	10.4–18.8	*29.9*–*36.9*	15.4	*32.4*	2.1	*1.6*
Claw 1 heights							
External base	18	6.9–16.1	*16.6*–*31.6*	10.5	*22.2*	2.2	*3.6*
External primary branch	18	15.3–32.1	*41.6*–*63.1*	23.4	*49.2*	4.6	*5.7*
External secondary branch	13	7.9–16.1	*21.0*–*31.0*	11.5	*24.2*	2.0	*2.7*
External base/primary branch (cct)	18	*36.4*–*57.3*		*45.1*		*5.9*	
Internal base	18	6.1–15.6	*17.5*–*30.6*	10.1	*21.2*	2.1	*3.3*
Internal primary branch	18	14.5–31.4	*40.2*–*60.5*	22.4	*47.1*	4.2	*5.1*
Internal secondary branch	13	6.1–15.3	*17.5*–*29.5*	11.0	*23.0*	2.2	*3.1*
Internal base/primary branch (cct)	18	*36.5*–*55.9*		*45.2*		*5.8*	
Claw 2 heights							
External base	13	7.6–18.5	*20.1*–*36.3*	12.1	*25.6*	2.8	*3.9*
External primary branch	14	16.5–35.5	*45.7*–*69.7*	25.9	*54.5*	5.9	*7.3*
External secondary branch	9	11.2–20.0	*25.9*–*39.3*	14.8	*29.7*	2.6	*4.2*
External base/primary branch (cct)	13	*40.3*–*54.7*		*47.1*		*4.6*	
Internal base	17	7.0–16.8	*17.9*–*33.0*	11.3	*23.9*	2.5	*3.5*
Internal primary branch	17	15.2–34.4	*42.1*–*67.6*	24.8	*52.3*	5.5	*7.2*
Internal secondary branch	14	9.4–17.2	*21.7*–*33.8*	13.4	*27.3*	2.0	*3.2*
Internal base/primary branch (cct)	17	*37.0*–*55.6*		*46.1*		*5.2*	
Claw 3 heights							
External base	14	6.8–19.2	*19.5*–*37.7*	12.1	*25.4*	2.9	*4.9*
External primary branch	14	15.7–37.1	*45.1*–*72.9*	26.4	*55.4*	5.2	*7.1*
External secondary branch	10	11.6–20.6	*25.6*–*40.5*	14.0	*28.9*	2.5	*4.3*
External base/primary branch (cct)	14	*38.5*–*55.7*		*45.7*		*5.8*	
Internal base	16	6.0–18.9	*17.2*–*37.1*	11.7	*24.8*	3.2	*5.0*
Internal primary branch	16	15.7–37.0	*43.8*–*71.9*	25.5	*54.0*	6.0	*8.2*
Internal secondary branch	11	11.0–19.5	*24.1*–*38.3*	13.7	*28.0*	2.4	*4.0*
Internal base/primary branch (cct)	16	*38.0*–*53.5*		*45.9*		*6.2*	
Claw 4 heights							
Anterior base	12	9.1–20.1	*22.8*–*39.5*	13.2	*28.5*	3.0	*4.1*
Anterior primary branch	12	27.2–49.4	*62.8*–*97.1*	37.3	*80.6*	6.7	*7.8*
Anterior secondary branch	11	11.0–24.4	*30.1*–*47.9*	15.9	*34.4*	3.8	*5.0*
Anterior base/primary branch (cct)	12	*30.6*–*44.9*		*35.4*		*4.1*	
Posterior base	13	10.3–21.9	*23.2*–*43.0*	15.6	*33.4*	3.6	*5.3*
Posterior primary branch	13	27.4–49.9	*63.3*–*98.0*	40.0	*85.8*	7.3	*8.9*
Posterior secondary branch	12	12.4–25.3	*30.4*–*49.7*	19.2	*40.3*	4.0	*5.9*
Posterior base/primary branch (cct)	13	*30.9*–*46.7*		*38.9*		*4.4*	

*Pt* values are given with italics.

#### 3.2.5. Eggs

**Figure 11:**
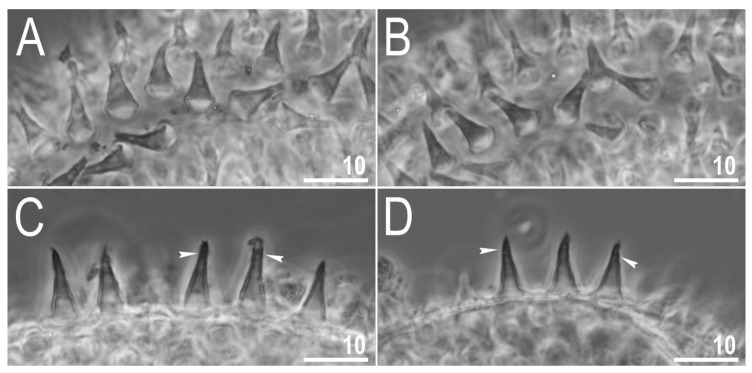
*Diaforobiotus hyperonyx***comb. nov.** (Maucci, 1982): Eggs seen in PCM: (**A**,**B**) Details of egg processes and surface under a ×1000 magnification; (**C**,**D**) midsection of the egg processes under a ×1000 magnification. Filled indented arrowheads indicate granulation on the distal portion of egg processes visible as dark dots and/or rough processes margins. Scale bars in μm.

**Figure 12:**
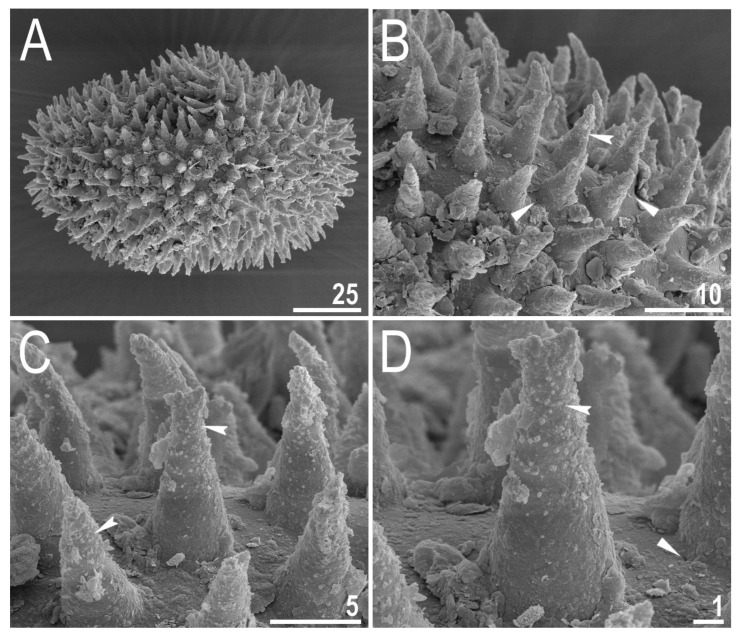
*Diaforobiotus hyperonyx***comb. nov.** (Maucci, 1982): Egg seen in SEM: (**A**) General view of the entire egg; (**B**–**D**) morphological details of egg surface and egg processes. Filled indented arrowheads indicate granulation on the distal portion of egg processes whereas filled flat arrowheads indicates micropores in the egg surface near processes bases. Scale bars in μm.

Laid freely, yellowish to light orange (Supplementary Materials SM.04), spherical with conical processes and smooth egg surface without areolation, reticulation or light-refracting dots (Figure 11A–D and Figure 12A–D). The process apices can sometimes exhibit faint nodular projection at the top (Figure 12B–D). Distal portions of the processes are covered by faint granulation: Dark dots of rough/jagged wall in the process midsection (PCM)/clear hemispheres (SEM) (Figure 11C,D and Figure 12A–D). The labyrinthine layer between the process walls as well as dark thickenings around process bases absent. Delicate micropores near the process bases rarely present and visible only in SEM (Figure 12B–D). Egg measurements and statistics are presented in Table 5.

**Table 5:** Measurements (in µm) of selected morphological structures of the eggs from the topotypic population of *D. hyperonyx* **comb. nov.** (Maucci, 1982) mounted in Hoyer’s medium; all three eggs were damaged in permanent slides, thus the diameter and number of processes on the egg circumference cannot be measured/counted; N—number of eggs/structures measured, RANGE refers to the smallest and the largest structure among all measured specimens; SD—standard deviation.

Character	N	Range	Mean	SD
Egg bare diameter	0	?	?	?
Egg full diameter	0	?	?	?
Process height	9	9.4–11.9	10.4	0.8
Process base width	9	4.0–5.5	4.7	0.5
Process base/height ratio	9	39–50%	46%	4%
Inter-process distance	9	2.7–4.9	3.6	0.7
Number of processes on the egg circumference	0	?	?	?

#### 3.2.6. Reproduction

**Figure 13:**
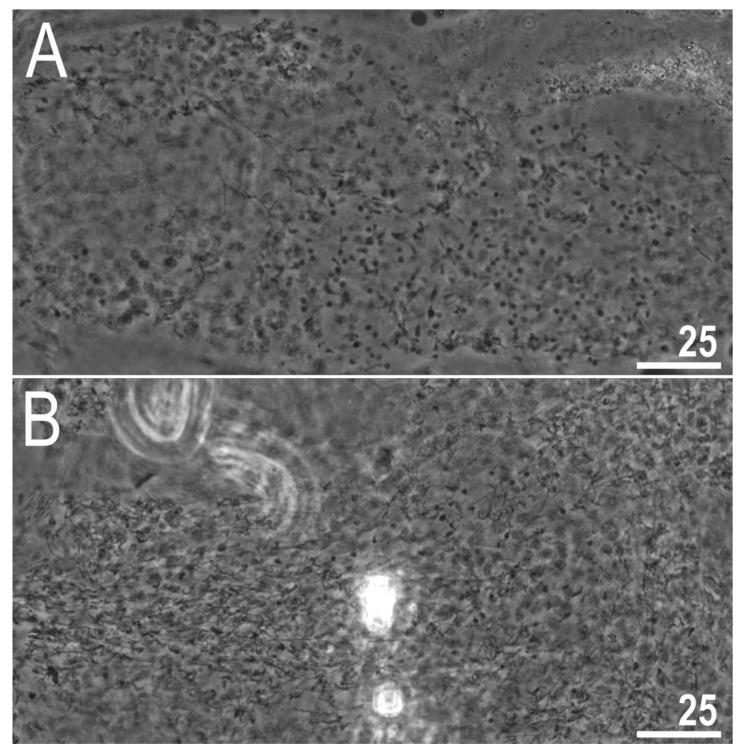
*Diaforobiotus hyperonyx***comb. nov.** (Maucci, 1982): Testes filled with spermatozoa: (**A**) A male from sample IT.339; (**B**) a male from sample IT.344. Scale bars in μm.

The examination of adults freshly mounted in Hoyer’s medium revealed testes filled with spermatozoa in each of the four examined samples, confirming the species to be dioecious (Figure 13A,B). Any other secondary sexual phenotypic characters, e.g., gibbosities on the hind legs in males, absent.

## 4. Discussion

Morphological information gathered in our integrative study on the newly discovered population, compared also with type specimens, fully support its identification as *Tenuibiotus hyperonyx* (Maucci, 1982) [40]. Based on the recovered phylogenetic position within the family Richtersiusidae as well as its phenotypic affinity to the genus *Diaforobiotus*, the species is proposed to be transferred to the later taxon. The proposed change requires amendments to the diagnosis of the family Richtersiusidae, which, among other characters, is now also defined by the presence of large teeth on all lunulae. Since *Diaforobiotus hyperonyx* **comb. nov.** exhibits teeth only in lunulae in the hind legs, the former character cannot be exclusive for the family. This further brings our attention to the two recent papers focusing on the phylogenetic relationships between four genera, namely *Diaforobiotus*, *Richtersius* Pilato & Binda, 1989 [94], *Adorybiotus* Maucci & Ramazzotti, 1981 [95] and *Crenubiotus*. Both these papers [11,27] were published at approximately the same time and came up with different interpretations of the relationship between the mentioned taxa. Guidetti et al. [11] studied the phylogenetic position of the newly discovered *Crenubiotus* species, also pinpointing its classification within the family Richtersiusidae, whereas Stec et al. [27] phylogenetically analyzed two different *Crenubiotus* and one additional *Adorybiotus* populations. Both studies recovered the sister relationships between clades *Adorybiotus* + *Crenubiotus* and *Murrayon* + *Dactylobiotus*, with *Richtersius* + *Diaforobiotus* being a sister clade to this entire cluster [11,27]. However, only Stec et al. [27] proposed splitting the family Richtersiusidae and erected the new family Adorybiotidae that comprises *Adorybiotus* and *Crenubiotus,* with the main distinctive character being the absence and presence of microplacoid in these families, respectively [27]. The phylogenetic analysis with increased taxa sampling conducted in our study yielded even higher support for the scenario proposed by Stec et al. [27], further confirming the validity of the family Adorybiotidae (Figure 1, Supplementary Materials SM.02). Finally, it would be worth discussing the obvious elongation of the claw primary branches in the *Diaforobiotus hyperonyx* **comb. nov.**, which make it distinct from all other *Diaforobiotus* populations recorded so far. This character could potentially constitute a clear diagnostic trait for a putative new genus, especially together with the recovered sister relationship between this species and remaining *Diaforobiotus* taxa (Figure 1). Nevertheless, we believe that, currently, the genus erection would be premature as the phylogenetic sampling of *Diaforobiotus* taxa is still scarce and the genus likely comprises at least several other species. Furthermore, the claw elongation in macrobiotids was recently reported to be caused most probably by the wet and icy environment [96]. The authors presented the phylogeny of the family Macrobiotidae demonstrating convergent evolution in claw elongation in the *Macrobiotus ariekammensis* complex and *Mesobiotus barabanovi* (Tumanov, 2005) [43], with both of them being deeply nested within their respective genera. The occurrence of *Diaforobiotus hyperonyx* **comb. nov.** in the high mountains (Dolomite Alps) further supports the hypothesis of environmental factors affecting the claw phenotypic changes in macrobiotids, constituting the third example of convergently evolving claws phenotypes within Macrobiotoidea.

**Figure 14:**
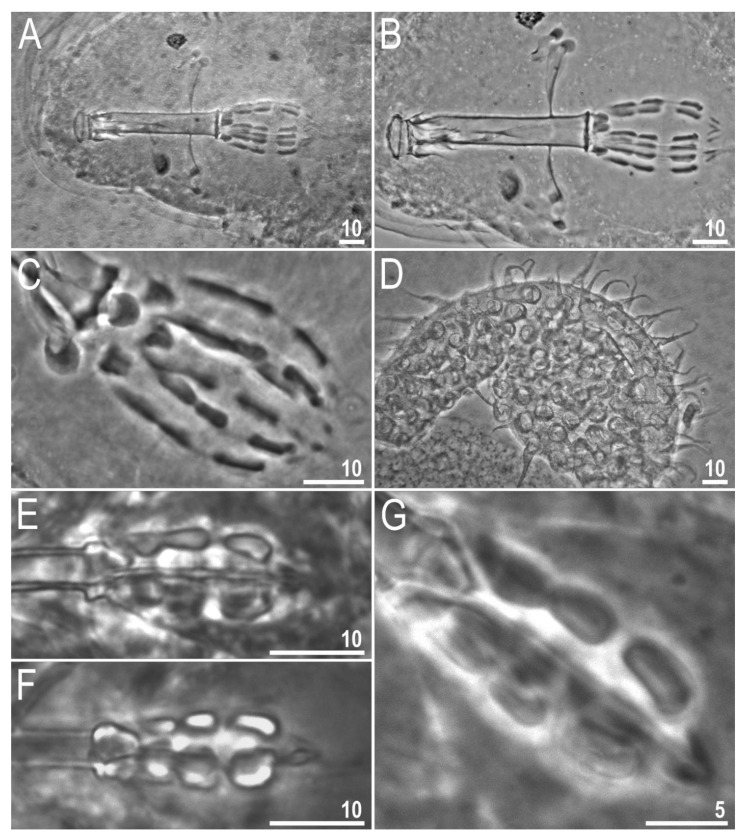
*Tenuibiotus willardi* (Maucci, 1982) and *Tenuibiotus bozhkae* Pilato, Kiosya, Lisi, Inshina & Biserov, 2011 types: (**A**,**B**) *T. willardi*: Bucco-pharyngeal apparatus of the holotype (the Pilato and Binda collection); (**C**) *T. willardi*: Placoids of the paratype (the Bertolani collection); (**D**) *T. willardi*: Egg (the Pilato and Binda collection); (**E**) *T. bozhkae*: Placoids of the holotype (the Pilato and Binda collection); (**F**,**G**) *T. bozhkae*: Placoids of the paratype (the Pilato and Binda collection). Scale bars in μm.

As mentioned in the Introduction, although the genus *Tenuibiotus* was recovered to be monophyletic in the phylogeny presented by Stec et al. [29], it was suggested to still be polyphyletic due to the morphological heterogeneity of the included taxa at that time. The mixed morphological characters that led to such a suggestion were: (i) The presence or absence of cuticular pores and (ii) varying numbers of placoids in the pharynx. After transferring *T. hyperonyx* to the genus *Diaforobiotus*, all remaining taxa currently recognized in the genus *Tenuibiotus* (13 species) exhibit a non-porous cuticle, which signifies that this trait is a solid and uniform diagnostic character of the genus. The nomenclatural and classification change proposed by us also provided the second uniform morphological trait that characterizes all members of the genus *Tenuibiotus*—the presence of a microplacoid in the pharynx. Regarding the number of macroplacoids, the majority of genus members exhibit two macroplacoids in the pharynx, excluding two species, which were reported to have three macroplacoids. These are *T. willardi* and *T. bozhkae*. The original description of the first one reports three or two macroplacoids to be present in the type population [48]. However, the re-examination of the holotype and paratype bucco-pharyngeal apparatuses confirmed the presence of two macroplacoids in the pharynx (Figure 14A–C). Since the name-bearing specimen exhibits this trait and it is considered important in the tardigrade classification, it should be considered the primary character state of the nominal species. The original description of the second species indeed reports three macroplacoids [89] and, at the same time, indicates morphological similarity with *Tenuibiotus ciprianoi* (Guil, Guidetti & Machordom, 2007) [97].

Interestingly, the latter species exhibits two macroplacoids in the pharynx, with the first one being extremely deeply constricted in the middle ([97]; Figure 3B). Although the quality of morphological documentation is much better in Guil et al. [97], the comparison of microphotographs presented in both these papers reveals that there is no obvious difference between them in this particular character ([89,97], especially the comparison of Figure 2B and Figure 3B, respectively). The examination of additional microphotographs of the holotype and paratype of *T. bozhkae* (Figure 14E–G) also did not allow us to indicate a clear difference between this species and *T. ciprianoi*. Thus, we consider *T. bozhkae* to possess only two macroplacoids in the pharynx, with the first one being deeply constricted. Therefore, we propose a third, uniform diagnostic morphological character for the genus *Tenuibiotus*, which is the presence of two macroplacoids in the pharynx.

## 5. Conclusions

Our work is yet another example of the great value of the integrated approach at a taxonomic and phylogenetic level when studying groups of small organisms such as meiofauna that are known to have a limited number of informative morphological characteristics. In this case, the revision of one enigmatic species and the discussion induced thereafter led to important amendments for other tardigrade taxa. We greatly endorse continuous studies on tardigrade taxonomy that implement the analyses of detailed phenotypic information tightly linked to genetic data. Surely, these will not only bring discoveries of new, exciting taxa but will also help to elucidate the trajectories of morphological evolution within this group of microscopic animals. 

## Figures and Tables

**Table 1:** Information on moss samples containing specimens of *T. hyperonyx* analyzed in the present study (A stands for animals and E stands for eggs).

Sample Code	Sample Type	Coordinates	Analyses		
			PCM	SEM	DNA
IT.339	moss	46°30′29.19″ N	6A + 0E	0A + 0E	1A + 0E
		11°49′41″ E			
IT.341	moss	46°30′26.9″ N	13A + 0E	0A + 0E	1A + 0E
		11°49′38.4″ E			
IT.344	moss + lichen	46°30′23.23″ N	18A + 2E	14A + 1E	1A + 0E
		11°49′31.8″ E			
IT.345	moss	46°30′23.23″ N	9A + 1E	0A + 0E	1A + 0E
		11°49′31.8″ E			

**Table 2:** Primers with their original references used for amplification of the four DNA fragments sequenced in the study.

DNA Marker	Primer Name	Primer Direction	Primer Sequence (5′-3′)	Primer Source
18S rRNA	18S_Tar_Ff1	forward	AGGCGAAACCGCGAATGGCTC	[54]
	18S_Tar_Rr1	reverse	GCCGCAGGCTCCACTCCTGG	
28S rRNA	28S_Eutar_F	forward	ACCCGCTGAACTTAAGCATAT	[55,56]
	28SR0990	reverse	CCTTGGTCCGTGTTTCAAGAC	
ITS-2	ITS2_Eutar_Ff	forward	CGTAACGTGAATTGCAGGAC	[13]
	ITS2_Eutar_Rr	reverse	TCCTCCGCTTATTGATATGC	
COI	LCO1490-JJ	forward	CHACWAAYCATAAAGATATYGG	[57]
	HCO2198-JJ	reverse	AWACTTCVGGRTGVCCAAARAATCA	

**Table 3:** GenBank accession numbers of the DNA sequences used for phylogeny reconstruction.

Species	18S rRNA	28S rRNA	COI	ITS-2	Sources
*Hypsibius exemplaris*	MG800327	MG800337	MG818724	MG800336	[58]
*Ramazzottius subanomalus*	MF001997	MF001998	MF001999	MG432819	[54]
*Bertolanius volubilis*	HQ604918	–	AY598769	–	[20,59]
*Bertolanius nebulosus*	GQ849023	–	–	–	[60]
*Eohypsibius nadjae*	HQ604921	–	–	–	[20]
*Minibiotus ioculator*	MT023998	MT024041	MT023412	MT024000	[35]
*Minibiotus pentannulatus*	MT023999	MT024042	MT023413	MT024001	[35]
*Tenuibiotus voronkovi*	KX810045	KX810049	KX810042	KX810046	[46]
*Tenuibiotus zandrae*	MN443040	MN443035	MN444827	MN443038	[47]
*Paramacrobiotus areolatus*	MH664931	MH664948	MH675998	MH666080	[17]
*Paramacrobiotus fairbanksi*	MH664941	MH664950	MH676011	MH666090	[17]
*Macrobiotus shonaicus*	MG757132	MG757133	MG757136	MG757134	[61]
*Macrobiotus caelestis*	MK737073	MK737071	MK737922	MK737072	[62]
*Xerobiotus pseudohufelandi*	HQ604989	–	AY598776	–	[20,59]
*Mesobiotus harmsworthi*	MH197146	MH197264	MH195150	MH197154	[63]
*Mesobiotus dilimanensis*	MN257048	MN257049	MN257047	MN257050	[64]
*Richtersius coronifer* NO.385	MH681760	MH681757	MH676053	MH681763	[18]
*Richtersius* aff. *coronifer* GR.008	MK211386	MK211384	MK214323–5	MK211380–1	[18]
*Richtersius* aff. *coronifer* IT.120	MH681761	MH681758	MH676054	MH681764	[18]
*Richtersius* aff. *coronifer* IT.317	MK211387	MK211385	MK214326–8	MK211382–3	[18]
*Richtersius* aff. *coronifer* PL.247	MH681762	MH681759	MH676055	MH681765	[18]
*Richtersius ziemowiti*	MT241891	MT241895	MT246504	MT241896	[7]
*Diaforobiotus islandicus* IS.042	MT812470	MT812461	MT808072	MT812597	[27]
*Diaforobiotus* sp. NO.386	MT812471	MT812463	MT808074	MT812598	[27]
*Diaforobiotus* sp. ID.517	MT812472	MT812462	MT808073	MT812599	[27]
***Diaforobiotus hyperonyx* IT.339**	**OM179853**	**OM179860**	**OM151287**	**OM179866**	**This study**
***Diaforobiotus hyperonyx* IT.341**	**OM179855**	**OM179861**	**OM151288**	**OM179868**	**This study**
***Diaforobiotus hyperonyx* IT.344**	**OM179852**	**OM179859**	**OM151286**	**OM179867**	**This study**
***Diaforobiotus hyperonyx* IT.345**	**OM179854**	**OM179862**	**OM151289**	**OM179869**	**This study**
*Murrayon dianae*	FJ435737	FJ435762	FJ435801	–	[65]
*Murrayon* cf. *pullari* IT.338	MT812477	MT812465	MT808080	MT812603	[27]
*Murrayon pullari*	GQ849026	–	–	–	[60]
*Dactylobiotus parthenogeneticus* FR.149	MT373694	MT373700	MT373804	MT374191	[34]
*Dactylobiotus parthenogeneticus* GB.003	MT373693	MT373699	MT373803	MT374190	[34]
*Dactylobiotus parthenogeneticus* PL.317	MT373695	MT373701	MT373805–6	MT374192	[34]
*Dactylobiotus selenicus* FI.073	MT812476	MT812466	MT808076	MT812602	[27]
*Dactylobiotus ambiguus*	GQ925676–7	–	–	–	Chen et al. (unpublished)
*Dactylobiotus ovimutans*	MT136805	–	MT132333	–	[66]
*Dactylobiotus octavi*	GQ849025	–	–	–	
*Crenubiotus* sp. GB.108	MT812473	MT812467	MT808077–8	MT812604–5	[27]
*Crenubiotus crenulatus* NO.429	MT812474	MT812463	MT808079	MT812606	[27]
*Crenubiotus ruhesteini*	MW074384–5, MW074387	–	MW074336–8	MW074367–8, MW074370	[11]
***Crenubiotus* sp. GL.001.01**	**OM179850**	**OM179857**	**OM151284**	**OM179864**	**This study**
***Crenubiotus* sp. GL.001.02**	**OM179851**	**OM179858**	**OM151285**	**OM179865**	**This study**
*Adorybiotus granulatus*	HQ604961–2	–	–	–	[20]
*Adorybiotus* cf. *granulatus* JP.008	MT812475	MT812464	MT808075	MT812600–1	[27]

Sequences obtained in this study are bolded.

## Data Availability

The author confirms that the data supporting the findings of this study are available within the article and its Supplementary Materials. The DNA sequences generated in this study are available in GenBank.

## Supplementary Materials

The following supporting information can be downloaded at: https://www.mdpi.com/article/10.3390/ani12030404/s1. SM.01. Best-fit partitioning schemes and models suggested by PartitionFinder; SM.02. Raw Bayesian and Maximum Likelihood trees given in Newick format; SM.03. Raw morphometric measurements of the topotypic population of *T. hyperonyx*. SM.04. Movie recording of an alive and gravid female of *T. hyperonyx*.
