# Supplementary material for: Reaching the Monophyly: Re-Evaluation of the Enigmatic Species Tenuibiotus hyperonyx (Maucci, 1983) and the Genus Tenuibiotus (Eutardigrada)
Source: Animals (Basel). 2022 Feb 8;12(3):404. doi: 10.3390/ani12030404 (PMC8833838; doi:10.3390/ani12030404)
Supplement: Supplementary file 1 [file animals-12-00404-s001.zip › SM.01.pdf]

BI:

Settings used

alignment : .\18S_28S_COI_ITS2.phy

branchlengths : linked

models : JC, K80, SYM, F81, HKY, GTR, JC+G, K80+G, SYM+G, F81+G, HKY+G, GTR+G, JC+I, K80+I, SYM+I, F81+I, HKY+I, GTR+I, JC+I+G, K80+I+G, SYM+I+G, F81+I+G, HKY+I+G, GTR+I+G

model_selection : bic

search : greedy

Best partitioning scheme

Scheme Name : start_scheme

Scheme lnL : -27683.3265991

Scheme BIC : 56601.7748511

Number of params : 154

Number of sites : 3042

Number of subsets : 6

Subset	Best Model	# sites	subset id	Partition names
1	SYM+I+G	1009	dbb1f009713256a64f462726a798bb1a	18S
2	SYM+G	832	c729b917f2760198563c64c7ad1a18e0	28S
3	HKY+G	220	68cb7022a49e378ae32b5449767de049	COI_pos2
4	SYM+I+G	219	bd8c5da9f24caf3f895ae5efeece8d69	COI_pos3
5	GTR+I+G	219	96289259977b5b0f7ffd7a1547dab639	COI_pos1
6	K80+I+G	543	50502d25058bd24193dccfc87b432c53	ITS2

ML:

Scheme BIC for different GTR models:

GTR	60167.5709396
GTR+G	56766.7606776
GTR+I	57932.8285487
GTR+I+G	56700.1250983
