# Supplementary material for: Reaching the Monophyly: Re-Evaluation of the Enigmatic Species Tenuibiotus hyperonyx (Maucci, 1983) and the Genus Tenuibiotus (Eutardigrada)
Source: Animals (Basel). 2022 Feb 8;12(3):404. doi: 10.3390/ani12030404 (PMC8833838; doi:10.3390/ani12030404)
Supplement: Supplementary file 1 [file animals-12-00404-s001.zip › SM.02.pdf]

BI tree:

((((Ber_neb:0.08869178999999994,Ber_vol:0.07073977000000009):0.5465504999999999,Eoh_nad:
0.3205600999999998):0.30051939999999977,(Ram_sub:1.232505,Hyp_exe:1.426674):0.230325500
00000018):0.1141553,((((((Ado_cf_gra_JP008_H1:9.769095000000672E-
4,Ado_cf_gra_JP008_H2:5.242566000001059E-
4):0.2680762000000001,(Ado_gra_1:0.023543440000000082,Ado_gra_2:0.023608850000000015):0
.03734802000000004):0.11290020000000012,(((Cre_cre_NO417:0.2715426999999999,(Cre_sp_GB1
08_H1:0.0014984160000000024,Cre_sp_GB108_H2:0.001925616999999935):0.0621313400000000
9):0.02491090000000007,(Cre_sp_GL_001_1:2.2864249999998698E-
4,Cre_sp_GL_001_2:2.2684899999991792E-
4):0.08020741000000009):0.2801247,(Cre_ruh_1:2.676617999999298E-
4,Cre_ruh_2:6.885948999999503E-4,Cre_ruh_3:2.6833460000008635E-
4):0.23734309999999992):0.13813690000000012):0.2104705,(((((Dac_amb_1:0.0211004200000000
1,Dac_amb_2:0.0504902599999999):0.05003430000000009,Dac_oct:0.02083626999999999,((Dac_
par_FR:2.6868320000006385E-4,(Dac_par_PL_H1:2.54002399999953E-
4,Dac_par_PL_H2:6.147564000000827E-
4):0.0018367200000000014):0.0035443390000000063,Dac_par_GB:0.0012145040000000051):0.05306
4189999999956,Dac_sel:0.09853460999999997):0.08629406999999989,Dac_ovi:0.17834149999999
993):0.10085999999999995,(Mur_cf_pul_IT338:0.09093163999999998,Mur_pul:0.02632181):0.123
10749999999993):0.11742359999999996,Mur_dia:0.7990218999999998):0.28797850000000014):0.
09864671999999963,((((Dia_isl_IS042:0.15239139999999995,Dia_sp_NO386:0.19228299999999998
):0.06684500999999998,Dia_sp_ID517:0.21057119999999996):0.07925399,((Ten_hyp_IT_339:2.621
1179999990897E-4,Ten_hyp_IT_345:2.593849000001036E-4):6.024539000000217E-
4,(Ten_hyp_IT_341:2.574051999999494E-4,Ten_hyp_IT_344:2.61312899999977E-
4):0.0016071870000000654):0.33269649999999995):0.12984529999999994,((((Ric_aff_cor_GR008_
H1:0.001133480000000002,(Ric_aff_cor_GR008_H2:0.0019931010000000526,Ric_aff_cor_GR008_H3
:0.002164983999999981):0.0015628960000000358):0.14362089999999994,((Ric_aff_cor_IT317_H1:
9.612848000000174E-4,Ric_aff_cor_IT317_H3:2.4776150000005437E-
4):0.004831277000000078,Ric_aff_cor_IT317_H2:0.003009891999999903):0.0995711100000007):
0.07074482000000004,(Ric_aff_cor_IT120:0.0035850969999999815,Ric_aff_cor_PL246:0.00142088
600000001):0.1379317):0.1642386,(Ric_cor:0.16960050000000004,Ric_zie:0.12399129999999992):
0.26401410000000003):0.17357849999999986):0.21079779999999992):0.1450241000000001,((((M
ac_cae:0.3532411,Xer_pse_1:0.3109275):0.2655299,Mac_sho:0.7506809999999999):0.4945094999
999997,(Meb_dil:1.0857,Meb_har:1.1523849999999998):0.9780044999999999):0.11665170000000
025,(((Min_ioc:0.37831919999999997,Min_pen:0.5318509):0.16155640000000004,(Ten_vor:0.0731
4529000000003,Ten_zan:0.057308520000000085):0.4180739):0.10334160000000003,(Pam_are:0.5
824257000000002,Pam_fai:0.6438873999999999):0.10861100000000001):0.30552519999999994):
0.20927929999999995):0.11415529999999992);((((Ber_neb:0.08869179,Ber_vol:0.07073977):0.546
5505,Eoh_nad:0.3205601):0.3005194,(Ram_sub:1.232505,Hyp_exe:1.426674):0.2303255):0.114155
3,((((((Ado_cf_gra_JP008_H1:9.769095E-4,Ado_cf_gra_JP008_H2:5.242566E-
4):0.2680762,(Ado_gra_1:0.02354344,Ado_gra_2:0.02360885):0.03734802):0.1129002,(((Cre_cre_N
O417:0.2715427,(Cre_sp_GB108_H1:0.001498416,Cre_sp_GB108_H2:0.001925617):0.06213134):0.0
249109,(Cre_sp_GL_001_1:2.286425E-4,Cre_sp_GL_001_2:2.26849E-
4):0.08020741):0.2801247,(Cre_ruh_1:2.676618E-4,Cre_ruh_2:6.885949E-4,Cre_ruh_3:2.683346E-
4):0.2373431):0.1381369):0.2104705,(((((Dac_amb_1:0.02110042,Dac_amb_2:0.05049026):0.05003
43,Dac_oct:0.02083627,((Dac_par_FR:2.686832E-4,(Dac_par_PL_H1:2.540024E-
4,Dac_par_PL_H2:6.147564E-

4):0.00183672):0.003544339,Dac_par_GB:0.001214504):0.05306419,Dac_sel:0.09853461):0.086294
07,Dac_ovi:0.1783415):0.10086,(Mur_cf_pul_IT338:0.09093164,Mur_pul:0.02632181):0.1231075):0.
1174236,Mur_dia:0.7990219):0.2879785):0.09864672,((((Dia_isl_IS042:0.1523914,Dia_sp_NO386:0.
192283):0.06684501,Dia_sp_ID517:0.2105712):0.07925399,((Ten_hyp_IT_339:2.621118E-
4,Ten_hyp_IT_345:2.593849E-4):6.024539E-4,(Ten_hyp_IT_341:2.574052E-
4,Ten_hyp_IT_344:2.613129E-
4):0.001607187):0.3326965):0.1298453,((((Ric_aff_cor_GR008_H1:0.00113348,(Ric_aff_cor_GR008_
H2:0.001993101,Ric_aff_cor_GR008_H3:0.002164984):0.001562896):0.1436209,((Ric_aff_cor_IT317
_H1:9.612848E-4,Ric_aff_cor_IT317_H3:2.477615E-
4):0.004831277,Ric_aff_cor_IT317_H2:0.003009892):0.09957111):0.07074482,(Ric_aff_cor_IT120:0.
003585097,Ric_aff_cor_PL246:0.001420886):0.1379317):0.1642386,(Ric_cor:0.1696005,Ric_zie:0.12
39913):0.2640141):0.1735785):0.2107978):0.1450241,((((Mac_cae:0.3532411,Xer_pse_1:0.3109275)
:0.2655299,Mac_sho:0.750681):0.4945095,(Meb_dil:1.0857,Meb_har:1.152385):0.9780045):0.11665
17,(((Min_ioc:0.3783192,Min_pen:0.5318509):0.1615564,(Ten_vor:0.07314529,Ten_zan:0.05730852
):0.4180739):0.1033416,(Pam_are:0.5824257,Pam_fai:0.6438874):0.108611):0.3055252):0.2092793)
:0.1141553);

ML tree:

(((Eoh_nad:0.14741491773838766,(Ber_neb:0.027783465975382837,Ber_vol:0.0213572786880354
97):0.25920330752230014):0.1486750797363472,(Ram_sub:0.6349283436079489,Hyp_exe:0.76153
51636230866):0.10315878834642112):0.055572024046004205,((((((Ado_gra_1:1.000000500073206
E-6,Ado_gra_2:1.000000500073206E-6):1.0000004999621837E-
6,(Ado_cf_gra_JP008_H1:0.0012112060928030077,Ado_cf_gra_JP008_H2:2.3308788930587188E-
4):0.1504980955661298):0.0527946892116855\1,((Cre_ruh_2:7.679249253188525E-
4,(Cre_ruh_3:1.000000500073206E-6,Cre_ruh_1:1.000000500073206E-6):1.000000500073206E-
6):0.15890658126046076,(Cre_cre_NO417:0.1495223606917968,((Cre_sp_GB108_H2:0.0035174696
029665053,Cre_sp_GB108_H1:0.0013663176568976532):0.04301134857936406,(Cre_sp_GL_001_1:
1.000000500073206E-6,Cre_sp_GL_001_2:1.000000500073206E-
6):0.0505427421609233б):0.010460858709026355):0.13163984209499602):0.06882354111937983)
:0.10455883287173329,(Mur_dia:0.38317013400081135,((Dac_ovi:0.11216828288997016,((Dac_pa
r_GB:1.000000500073206E-
6,(((((Dac_amb_2:0.012843924812830365,Dac_amb_1:1.000000500073206E-
6):0.012851007862592412,Dac_par_PL_H2:1.000000500073206E-6):6.576281490285885E-
4,Dac_par_PL_H1:1.000000500073206E-6):1.000000500073206E-6,Dac_oct:1.000000500073206E-
6):0.002712401198256442,Dac_par_FR:1.000000500073206E-
6):0.006881772500100025):0.042713239747613985,Dac_sel:0.07973693515003366):0.0497434608
2891001):0.047450612481807486,(Mur_pul:1.000000500073206E-
6,Mur_cf_pul_IT338:0.029563762284088746):0.09044423872931595):0.05449980904736062б):0.13
989312388124453):0.04581235279039375,((((Ten_hyp_IT_341:1.000000500073206E-
6,Ten_hyp_IT_344:1.000000500073206E-
6):0.0025526322146107727,(Ten_hyp_IT_345:1.000000500073206E-
6,Ten_hyp_IT_339:1.000000500073206E-6):1.000000500073206E-
6):0.17819268254563525,((Dia_sp_NO386:0.10147353295962913,Dia_isl_IS042:0.0881777736893б
841):0.034134312360666996,Dia_sp_ID517:0.12245477743392597):0.03603179428561898):0.0622
692069681543,(((Ric_aff_cor_PL246:0.0014719760653552072,Ric_aff_cor_IT120:0.00553398992046
4055):0.07880732374110233,(((Ric_aff_cor_IT317_H1:0.0012320728867720732,Ric_aff_cor_IT317_

H3:1.000000500073206E-
6):0.006578010801883694,Ric_aff_cor_IT317_H2:0.004287402245703231):0.054851042495164704,
(Ric_aff_cor_GR008_H1:1.000000500073206E-
6,(Ric_aff_cor_GR008_H2:0.0030938106455011827,Ric_aff_cor_GR008_H3:0.003155418133578447)
:0.0031000135565417697):0.07555680808492737):0.03981379117080053):0.07609518090173484,(
Ric_cor:0.09274697628387285,Ric_zie:0.07573240280267657):0.14150369313478328):0.086849341
0860457):0.0955259643369164):0.05942914623569795,(((Min_pen:0.2904219813409984,Min_ioc:0
.18233803983758368):0.07062038471148069,((Pam_fai:0.3150123360692493,Pam_are:0.28874578
692612274):0.06126574507573934,(Ten_zan:0.035696219183018685,Ten_vor:0.045160566972080
82):0.21607945260335193):0.030400666130638188):0.13927310796167336,((Mac_sho:0.37536182
97584308,(Mac_cae:0.19635232379646272,Xer_pse_1:0.18358115411591858):0.125318018717072
6):0.2437870266640484,(Meb_har:0.5806984318118116,Meb_dil:0.576782267535227):0.49461760
252343523):0.04353351392911975):0.09709932897924078):0.05557202404600408);
